# The understanding of research ethics at health sciences schools in Jordan: a cross-sectional study

**DOI:** 10.1186/s12909-020-02040-5

**Published:** 2020-04-21

**Authors:** Nafez Abu Tarboush, Zaid Alkayed, Karem H. Alzoubi, Wael K. Al-Delaimy

**Affiliations:** 1 Department of Biochemistry and Physiology, School of Medicine, The University of Jordan, Amman, 11942 Jordan; 2 Internal Medicine Unit, Psychiatry Division, Jordan University Hospital, Amman, Jordan; 3 Department of Clinical Pharmacy, Jordan University of Science and Technology, Irbid, Jordan; 4 Department of Family Medicine and Public Health, University of California San Diego, San Diego, CA USA

**Keywords:** Awareness, Ethical principles, Informed consent, Research ethics committees, Conflict of interest, Authorship, Ownership

## Abstract

**Background:**

Research ethics is required for high-quality research that positively influences society. There is limited understanding of research ethics in Middle Eastern countries including Jordan. Here, we aim to investigate the level of understanding of research ethics principles among health sciences faculty members in Jordan.

**Methods:**

This is a cross sectional study where faculty members from the University of Jordan were surveyed for their knowledge and, attitude of research ethics principles. The study was conducted in the period between July 2016 to July 2017 using a customized-design questionnaire involving demographic data and participants’ contributions toward research, and assessment of participants’ knowledge, belief and attitude towards research ethics. Different question-formats have been used including multiple-choice, yes or no, and a four point Likert-type questions. Obtained responses were tabulated according to gender, academic-rank, and knowledge about research ethics principles.

**Results:**

The study had a response rate of 51%. Among the 137 participants of this study, most (96%) were involved in human and animal research, yet, only 2/3 had prior training in research ethics. Moreover, 91% believed that investigators should have training in research ethics and 87% believed that there should be a mandatory postgraduate course on that. The average correct scores for correct understanding of researchers towards research ethics was 62%. Yet, there were some misconceptions about the major ethical principles as only 43% identified them correctly. Additionally, the role of research ethics committees was not well understood by most of the respondents.

**Conclusions:**

Although there is acceptable knowledge about research ethics, discrepancies in understanding in research ethics principles seems to exist. There is a large support for further training in responsible conduct of research by faculty in health sciences in Jordan. Thus, such training should be required by universities to address this knowledge gap in order to improve research quality and its impact on society.

## Background

The field of research ethics is a complex and ever-changing topic. Between international and national guidelines down to individual institutional Research Ethics Committees (RECs) requirements, it is an ever-pressing necessity for researchers to familiarize themselves with the general ethical concepts and information needed to guarantee research approval and publication and avoidance of retractions.

Various studies have been conducted to assess knowledge and awareness of research ethics among members in different institutions with results showing up to 11% of investigators agreeing that it is acceptable to fabricate data if it would improve the outcome of a study [1, 2]. A study by Weston K. M.et al. in two medical schools in Australia demonstrated varying attitudes among academic staff and clinicians towards various ethical issues in conducting research [3]. Additionally, a study form the Middle East showed that 28% of researchers in the region did not obtain ethical clearance for their proposals [4]. El-Dessouky H. F. et al. conducted a study in two dental schools in Saudi Arabia and Egypt; where they demonstrated that less than half of respondents were familiar with research ethics principles, and less than a third were familiar with the functions of RECs [1].

Several research ethics documents currently exist including the Nuremberg code, the Declaration of Helsinki, the Belmont Report, and the International Ethical Guidelines for Biomedical Research Involving Human Subjects. Though there is not a complete consensus among these documents, they still provide key ethical principles for research involving human subjects. Moreover, several countries currently refer to them in their own national guidelines [5, 6]. A study of the current research ethics guidelines in Middle Eastern countries revealed varying degrees of development and structure among them, with some countries not having national guidelines at all, and others having up to three guidelines [6].

Jordan has been the first Arab country to enact clinical trials research regulations. The Jordanian clinical trials law is based on Declaration of Helsinki, and it governs all clinical trials research in the country [7] though it has some shortcomings, such as regarding guidelines concerning research involving children [8, 9]. Recent studies have assessed knowledge and attitudes towards research ethics in various groups. A study has demonstrated favorable attitudes of researchers towards RECs [10]. Another study found that very few resident doctors had knowledge of The Declaration of Helsinki, and only 36% had prior clinical research experience [11]. Efforts to increase medical students’ and residents’ exposure to research are being implemented at University of Jordan (JU) medical school with the implementation of a mandatory research project for students, and a mandatory publication for residents’ prior graduation. However, mandatory research ethics courses or workshops are not being incorporated yet.

JU is the first public university in Jordan, it has currently the largest number of faculty members and students, and there have been guidelines and a research ethics committee since late 1990s. It is clear from the literature that current research ethics education and regulations have not fully translated into proper knowledge and attitudes throughout the world. This study aims to assess the current understanding of research ethics among faculty members of health sciences schools (HSS) at JU as an example for the country and the region.

## Methods

### Study design

A cross-sectional survey was conducted in the period between July 2016 to July 2017. The questionnaire collection was in person which decelerated the process. The research assistants (trained students) were approaching professors at their offices during their office hours. Some professors were on sabbatical or unpaid leaves while others were unavailable or busy when research assistants approached them which mandates frequent visits. Also, there was some dependence on students’ schedule. Furthermore, the study began in the summer when many professors were on summer vacation which stalled the process. Despite the prolonged duration in the collection process, it is unlikely for the results or data interpretation to be affected due to the absence of any formal training program during that period.

The questionnaire of the study was designed to better fit the target group (Additional file 1). It was prepared after reviewing relevant literature [1, 4, 10, 12, 13], and taking the opinions and feedback of faculty in both medical and ethics fields. It was a 4-page questionnaire; the first section inquired about demographic data and participants’ contributions toward research. The second section assessed participants’ Knowledge, belief and Attitude towards research ethics. Different question-formats have been used; multiple-choice questions were participants can choose more than one answer, yes or no questions, and a four point Likert-type questions (Agree, Disagree, Neutral, and I don’t Know). The questions ranged from asking about knowledge regarding ethical guidelines and applications, and requirements for ethics training, mandate of research ethics committees, requirement for informed consent, how to report research misconduct, who owns the data, familiarity with research ethics misconduct terms, and membership of research ethics committees (see tables and figures for description of these questions). Questionnaires which were not fully completed were disregarded from analyses.

### Study participants

Members of the health sciences faculty (Lecturers, Assistant Professors, Associate Professors, and Professors) from four HSS (Medicine, Dentistry, Pharmacy, and Nursing) were approached at JU, Amman, Jordan. HSS were chosen for many reasons where convenience in obtaining the questionnaires played a role, and English language proficiency played another important role since the media of instruction and teaching at HSS is English, while it varies at other schools. Any faculty member who is appointed by JU as a full-time employee and can apply for research grants in these four schools was considered eligible which includes all faculty members (367 members including those on sabbatical or unpaid leaves). JU is considered the oldest university in Jordan with the largest number of faculty members (1443 members) and students (≈ 50,000 students). Lists of faculty member names were obtained and were approached by research assistants multiple times till they meet the faculty member in person without any prior information about their rank, experience, or their previous ethical training. Faculty who were approachable were 268 members during data collection and those who have fully completed the questionnaire were 137.

### Data analyses

Data were collected and analyzed using EXCEL 16 and GraphPad Prism version 5. Descriptive statistics were used to summarize the data. Correct responses for each question (Additional file 1) were recorded as percentages out of total answers. Likert-type questions were formulated to insure respondents’ who are able to strongly pinpoint the correct knowledge or attitude among researchers. Accordingly, the four-point scale has been transformed into a binary scale where the right answer (agree or disagree) was considered correct while other answers where considered incorrect, then percentages of correct answers were calculated and reported. Chi-square test was used for cross-tabulation with gender, type of health school, academic rank, and knowledge regarding publication ethics. Values of *p* <  0.05 were considered to be statistically significant.

## Results

### Respondents’ demographics and experience

Faculty members (*n* = 137) were included in the study with a 51% response rate. There were 74 (54%) and 63 (54%) males and females, respectively. Table 1 shows their demographic characteristics and their distribution according to academic rank, and research experience. Mean age of the participants was 42.2 ± 11.7 years. Most respondents were engaged in research projects provided by their publications’ record during the last five years. The vast majority of respondents were involved in human or animal research (*n* = 131, 96%). However, only two thirds of them (*n* = 87, 64%) had prior research ethics course or training. No statistically significant association was detected linking prior research ethics training with the specific country or region where the respondent had obtained his/her highest academic degree.

**Table 1:** Characteristics of study participants

Variable	N (%) or Mean ± SEM
Age (years)	42 ± 5.5
Gender (Male, Female)	74, 63 (54, 46)
Academic rank	
Lecturer	39 (28)
Assistant Professor	41 (30)
Associate Professor	30 (22)
Professor	27 (20)
Years of experience at the institution	10.0 ± 0.9
Number of publications in the last 5 years	7.4 ± 0.7
Faculty performing projects involving human or animal subjects	96 (70)
Prior research ethics training or exposure	64 (47)

### Importance of research ethics field

Majority of respondents (87%) thought research is bound by ethics and morality. Further, 91% of respondents thought that all investigators should have training in research ethics, and 87% of them believed that there should be a mandatory postgraduate course on research ethics (Table 2). Nevertheless, there were misconceptions about the purposes that research ethics should serve among some of the researchers. For example, 25% of respondents thought that one of the goals of research ethics is to maximize publication quantity, and 15% of them thought that securing personal finances is one of the goals.

**Table 2:** Knowledge of participants about research ethics with the percentage agreeing to the questions listed in the table and the statistical significance of difference according to gender, academic rank and the faculty they belonged

No.	Item	% of correct responses	*P* value		
			Gender	Academic Rank	School
1	All research ethics guidelines apply to all societies and cultures	25	0.81	0.24	0.44
2	Research ethics course should be mandatory in postgraduate programs	87	0.56	0.91	0.22
3	All investigators of human and animal studies should have training in research ethics	91	0.14	0.48	0.01 *
4	Not all participants comprehend research projects well. Accordingly, there is no need to provide them with details	78	0.29	0.15	0.15
5	There is no need to obtain informed consent to do research on blood samples already withdrawn for clinical tests	70	0.56	0.30	0.00 ***
6	There should be an REC at each university	88	0.23	0.22	0.49
7	Only human subject research must be reviewed by an REC	69	0.26	0.07	0.27
8	Review by an REC would delay research projects and make it harder for the researcher to perform it	29	0.24	0.39	0.05
9	If there is a scientific committee for reviewing research, there is no need for an REC	78	0.29	0.72	0.84
10	Members of the REC should be at least professors with high authority in the university	64	0.08	0.34	0.59
11	There is no need for child approval in research if they are less than 15 years as long as parents are consenting	57	0.20	0.13	0.00 **
12	Retrospective studies are exempt from informed consent	39	0.88	0.84	0.05
13	The researcher (by him/herself) can decide that no informed consent is needed if the research is a retrospective study (data already collected)	56	0.63	0.82	0.20
14	Informed consent should be always written	34	0.95	0.23	0.05

*, **, *** refers to *p*-values < 0.05, < 0.01, < 0.001, respectively

### Knowledge of research ethics, informed consent, and RECs role

**Fig. 1:**
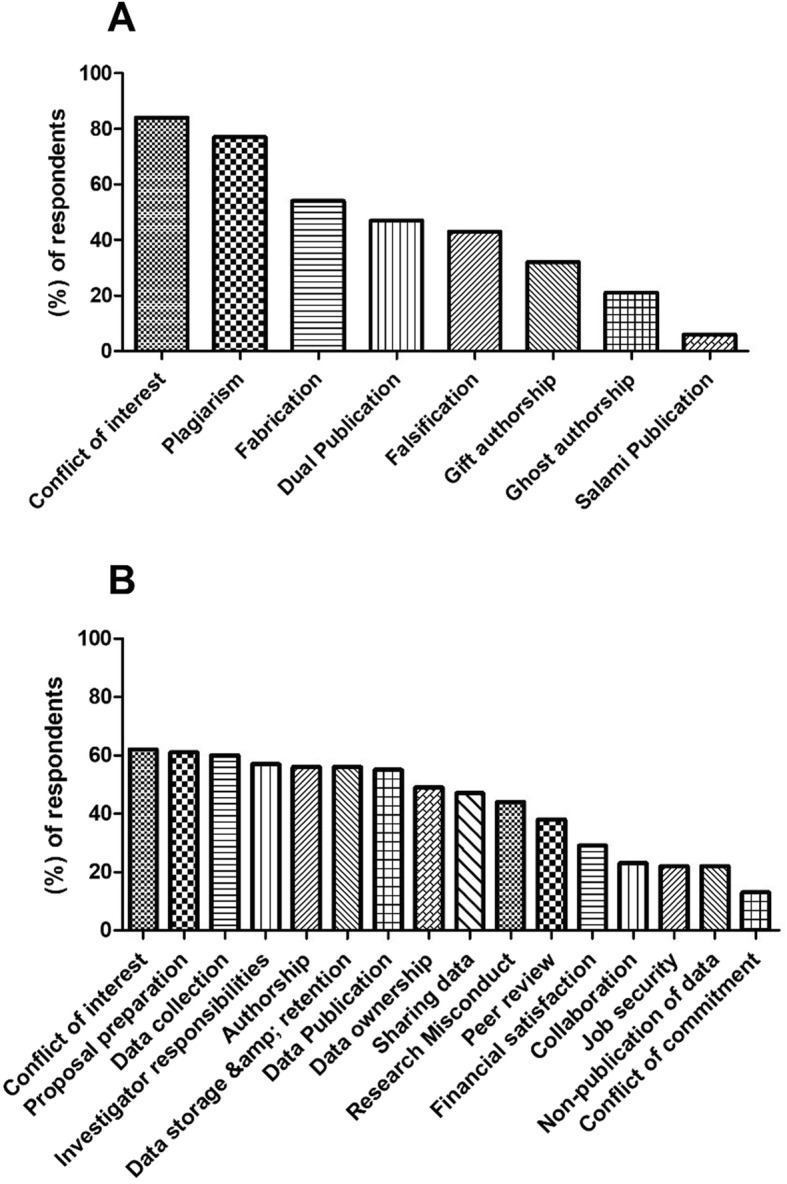
Respondents’ familiarity with research ethics terms and their awareness of specific existing research ethics guidelines. Each column represents the percentage of respondents who considered themselves familiar with certain research ethics terms (x-axis) (Additional file 1, KAP section, Q7) (**a**) and respondents who considered themselves knowledgeable if certain terms (x-axis) have guidelines in research ethics (Additional file 1, KAP section, Q8) (**b**)

60% of respondents considered gaining public trust as a major theme of research ethics. Moreover, 75% of participants considered research ethics as flexible and which can be tailored towards different societies and cultures. In addition, 93% of participants claimed familiarity with the major ethical principles. However, only 43% of them could specifically point out the three major ethical principles (Autonomy, Justice, and Non-maleficence). Figure 1 illustrates the familiarity of respondents with certain terms in research ethics and if guidelines exist to regulate-them. Participants were most familiar with Conflict of Interest (COI) and plagiarism (Fig. 1a). However, their familiarity with other terms was less than 50%. Additionally, COI was on top of the list regarding participants’ knowledge of existence of guidelines (62%), while approximately 50% of participants had knowledge that guidelines existed for other topics (Fig. 1b).

The four point Likert-type questions were directed to assess the general knowledge, attitude, and practice (KAP) of the respondents (Table 2). The average percentage of correct answers to the 14 questions asked was 62%, and the percentage of correct answers for each question was as shown in Table 2. Chi-square analysis of respondents’ KAP did not detect any statistically significant association between answering correctly and the participants’ gender or academic rank. However, results showed a statistically significant association between being in a specific school and answering correctly for a number of the KAP items (Table 2).

**Fig. 2:**
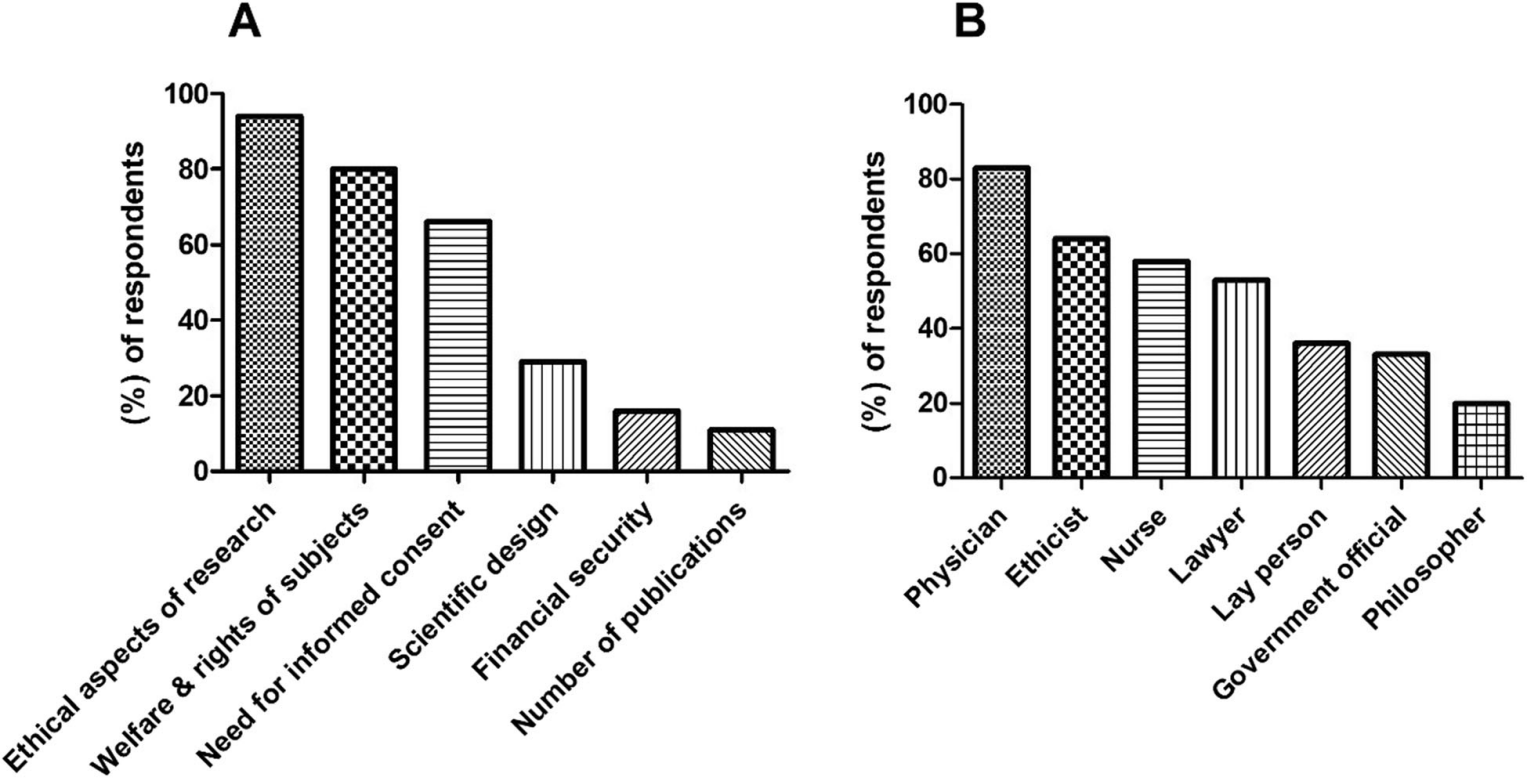
Perception of RECs role and who should be a member on the committee among respondents. Each column represents the percentage of respondents perceived REC role as the roles assigned in the x-axis (Additional file 1, KAP section, Q11) (**a**) and their perception of who should be a member of an REC (Additional file 1, KAP section, Q12) (**b**)

Figure 2a demonstrates respondents’ knowledge of RECs roles. A high percentage of respondents thought of REC as a committee to oversee the ethical aspects of research, and to protect the welfare of research subjects (94, 80%, respectively). Nevertheless, two-third of participants thought of it as a committee to decide if there is a need for informed consent, and only 29% of participants thought an REC has the right to interfere with the scientific design of the study.

Responses to the question of who should be a member in RECs are displayed in Fig. 2b. A high percentage thought a physician, an ethicist, and a nurse should be members of RECs (83, 64, and 58% respectively). On the other hand, approximately one third or less thought a layperson, a government official, or a philosopher are needed as members in RECs.

### Data retention, ownership, and authorship

Almost three quarters (74%, *n* = 102) retained documents for over three years, while less than 7% kept data for less than one year. Respondents also had the choice between sole or shared ownership of data. Approximately, 18% thought of students as a member or the sole owner of the data while 48, 43, and 12% thought of the Primary Investigator (PI) and collaborators, School or University, and funding agency, respectively, as members or sole owners of the data (Table 3).

**Table 3:** Responses of participants regarding who should own the data of a study

Body who should have data ownership	%
PI and collaborating researchers (not including students)	37
School or university of researcher/s	33
The graduate student/s (if any)	10
The granting agency	4
Shared^a^	16

^a^Response was shared among any combinations of PI, collaborating researchers, school or university of researcher/s, the graduate student/s, and the granting agency

Results revealed that 39% (*n* = 53) of respondents answered that as an author you need to be an active part of three processes in any research project; project design, data analysis, and manuscript preparation. Other respondents considered a single activity out of the three qualifies for authorship. Still others, considered further activities such as data collection and being the PI who obtained the funding qualify for an authorship. This indicates the lack of specific adopted guidance of authorship qualification. In addition, analysis found no association of the answers with the academic rank of the respondents.

## Discussion

This study showed contradiction between knowledge and attitude regarding research ethics. There was a deficiency in knowledge regarding the subject of research ethics among study participants. This, however, did not correlate with the respondents’ subjective reports of previous ethics training and exposure. On the other hand, 36% of respondents did not have prior research ethics training. Although this result is comparable to other studies from high-income and low-and-middle-income countries [1, 12, 13], the majority of respondents (93%) performed research projects involving humans or animals, which mandates higher level of research ethics training.

Current results revealed a high appreciation of the respondents to the autonomy of research participants (Table 2, items 4 & 5). However, specific attitudes toward informed consent (Table 2, items 11–14) revealed varying attitudes of respondents in comparison with the published literature [1, 12–17]. Specific issues on informed consent are growing rapidly in the field of research ethics; e.g. the issue of written consent. Approximately, two thirds of respondents believed that informed consent should always be written. Whereas, in fact, the consent should be informed and culturally appropriate regardless of the method of documentation [18, 19].

Exempting retrospective studies from informed consent is another issue that has been answered correctly by less than half of participants. It is the RECs that are authorized to make that decision, not the investigator(s).

An important point to stress is the practical and everyday exposure of the participants to research ethics issues at their institutions. In the two questions that asked about familiarity of respondents with research ethics terms and if international guidelines are applied in research ethics topics, the highest percentage scored in both items was for COI. This could be related to manuscript submission process where COI process has to be declared. With that said, in addition to formal ethics training, the implementation of ethical considerations at the level of the institution may provide practical exposure of faculty members to ethical issues that solidifies the theoretical information they gained beforehand.

Respondents had some contradicting understanding and perception about the role of REC, as the majority (88%) believed that RECs should be created in each university, yet, one third of the respondents believed that project reviewing by an REC would delay research projects approval and/or funding process. These findings corresponds with previous studies reporting excessive bureaucracy caused by RECs in Jordan [10] and other Western and Mediterranean countries [1, 20]. This might be related to a perception of respondents about delay rather than an actual delay. Therefore, proposals that aim to ease the process of project approval without compromising ethical or scientific regulations are a necessity, both locally and internationally.

In terms of data ownership; this varies by institutions and usually require compliance with the funding agency or university requirements. Sharing data has become the common rule globally [21]. However, 84% of respondents chose a single owner, with students having the least rights to ownership. This suggests the idea of single ownership, which should, theoretically, place strict rules on authorship qualifications. However, this was not reflected in current data as many respondents considered a single activity such as data collection qualifies for authorship. With only 39% of respondents correctly identifying the proper authorship qualification, it seemed more plausible that authorship is seen as a mean for academic promotion rather than being based on merits and project participation. This does in fact echo the argument of “publish or perish” environment [22].

Although only 51% responded to the current survey, this is similar to other such surveys done elsewhere [23, 24]. This study is potentially underestimating the gap between knowledge and attitude as most of those who responded would likely be interested in the topic and follow ethical guidelines, while those who did not might be less informed and therefore less interested in such a survey. It might also be due to the non-participants being busy clinicians or non-researchers and in such case the results are still an underestimation of the gaps in research ethics knowledge. An appropriate monitoring approach by universities can include mandatory surveys of all researchers applying for REC approval.

Results reported in the current study indicate a possible gap between theoretical knowledge and real world application among participants. This could be reflective of an underlying approach to research ethics being mostly an academic exercise. Moreover, this view is consistent with the current publish-or-perish environment. Academics are pushed to publish research focused on quantity rather than quality, and as such ethical considerations and understanding take a lower position in the researchers’ priorities.

### Study limitations, strengths, and future directions

This study is bound to several limitations. First, faculty members who were enrolled and completed the questionnaire not necessarily reflects the entire faculty KAP toward research ethics provided by the response rate of 51%. Second, despite that JU is the largest university with the biggest number of faculty members, this study still involved only a single institution in Jordan, which could limit the generalizability of our results. Further studies that involve other schools at JU and other universities in Jordan are encouraged and necessary to evaluate the baseline of research ethics KAP among our researchers. Furthermore, we did not investigate the type of previous training of researchers, which might impact their KAP toward research ethics and give a clearer vision of what is recommended to implement in the future at the institution. On the other hand, the study revealed some interesting points. Firstly, there appears to be an acceptance of RECs among the faculty, which is in accordance to what researchers believe at another institution in Jordan [10]. Second, there seems to be a need for formal education in research ethics among all faculty levels, which is also in accordance a previous study of medical residents’ beliefs in Jordan [11]. Finally, consideration for qualitative or mixed methods methodology in future would be sensible as the qualitative components would be highly informative and instructional for further progress of any training.

## Conclusions

Although researchers seem to have acceptable knowledge about research ethics, discrepancies in understanding research ethics principles seems to exist. Thus, formal mandatory training in research ethics with further evaluation and research should be considered for all research-involved personnel at different undergraduate and postgraduate level. Furthermore, institutional regulations and oversight of research projects are a cornerstone to prevent misconduct. More stringent processes of approving research with constructive feedback from knowledgeable reviewers may increase awareness and knowledge indirectly by forcing researchers to adhere to ethical guidelines or face rejections.

## Supplementary information

**Additional file 1.**

## Data Availability

The datasets used and/or analyzed during the current study are available from the corresponding author on reasonable request.

## Abbreviations

COI: conflict of interest
HSS: health sciences schools
JU: The University of Jordan
KAP: knowledge, attitude, and practice
PI: primary investigator
REC: research ethics committee
