# Supplementary material for: The understanding of research ethics at health sciences schools in Jordan: a cross-sectional study
Source: BMC Med Educ. 2020 Apr 21;20:121. doi: 10.1186/s12909-020-02040-5 (PMC7175529; doi:10.1186/s12909-020-02040-5)
Supplement: Supplementary file 1 — Additional file 1. [file 12909_2020_2040_MOESM1_ESM.pdf]

Appendix A

Demographic and research experience				
1. Age		2. Gender	M	F
3. Rank	Lecturer	Assistant Prof.	Associate Prof.	Full prof.
4. Faculty	Medicine	Dentistry	Pharmacy	Nursing
5. Projects performed involves *(check all that apply)*	Surveys	Cells	Animals	Human (experimental)
**Knowledge, Attitude and Practice (KAP) of research ethics**				
1. Research ethics is important	✓ Strongly agree	✓ Agree	Disagree	Strongly disagree
2. If agree, why do you think research ethics is important? *(check all that apply)*	✓ Promoting research best practices		✓ Create open communication	
	Securing personal finances		✓ Gaining public trust	
	Maximizing publication quantity		✓ Gaining governmental trust	
	✓ Protecting participants		✓ Gaining private sector trust	
	✓ Sharing ideas		✓ Promoting morality	
3. Is research irrelevant to ethics and morality?	Yes		✓ No	
4. Have you been exposed to formal research ethics training (course, workshop, etc.)?	Yes		No	
5. Are you familiar with the basic major ethical principles of research?	Yes		No	

✓ Correct answers and/or what is considered good practices

Appendix A

6. The following is a major research ethical principle *(check all that apply)*	✓ Autonomy: (freedom of participants to decide what to do)	✓ Justice: (the fair selection of research participants)
	Conflict of interest: (deciding when to participate or not in research projects when you have a conflict)	Collaboration: (deciding how research team relations should be)
	Authorship: (deciding who deserves authorship and who does not)	✓ Non-maleficence: (non-harming or inflicting the least harm possible to reach a beneficial outcome)

7. Which of the following terms are you familiar with in research? *(check all that apply)*	Plagiarism	Fabrication	Falsification	Ghost authorship
	Gift authorship	Conflict of interest	Dual publication	Salami publication

8. Which of the following terms do you think has guidelines in research ethics? *(check all that apply)*	✓ Proposal preparation	✓ Investigator responsibilities	Financial satisfaction	✓ Data collection
	✓ Research misconduct	✓ Data publication	✓ Non-publication of data	✓ Data storage and retention
	✓ Authorship	✓ Conflict of interest	✓ Conflict of commitment	✓ Collaboration
	✓ Sharing data	✓ Data ownership	✓ Peer review	Job security

9. For how long do you keep all your projects' documents?	< 1 year	1-3 years	✓ 3-5 Years	✓ > 5 years

10. From your perspective, who owns the data of a project (you can choose more than one answer)?	The student	The PI ± collaborators	The school or university	Granting agency

11. What do you think is the role of a research ethics committee? *(check all that apply)*	✓ To review ethical aspects of research	✓ To review the scientific design of research
	✓ To determine if informed consent is needed in research of human subjects	✓ To protect the welfare and rights of research participants
	To ensure financial security of researcher	To ascertain number of publications that should come out of the project

Appendix A

<table>
<tr><td rowspan="2">12. Who of the following persons do you think is justified to be a member of an REC?
(check all that apply)</td><td>✓ Physician</td><td>✓ Nurse</td><td>✓ Ethicist</td><td>✓ Philosopher</td></tr>
<tr><td>✓ Lawyer</td><td colspan="2">✓ Any regular person (lay person)</td><td>Government official</td></tr>
<tr><td>13. Which of the following(s) is (are) considered guidelines in research ethics?
(check all that apply)</td><td>✓ Nuremberg Code</td><td>✓ Declaration of Helsinki</td><td>✓ Belmont Report</td><td>✓ Council of the International Organizations of the Medical Sciences (CIOMS)</td></tr>
<tr><td rowspan="2">14. Which of the following activities accomplished by research personnel deserves authorship?
(check all that apply)</td><td>Project design only</td><td>Data collection only</td><td>Manuscript writing only</td><td>Statistical analyses only</td></tr>
<tr><td>Data analysis only excluding statistical analyses</td><td colspan="2">Being the senior Investigator who brought the funding</td><td>✓ Project design & data analysis & manuscript writing</td></tr>
</table>

Appendix A

Please, comment on the following statements:

1. All research ethics guidelines apply to all societies and cultures	Agree	✓ Disagree	Neutral	I don't know
2. Research ethics should be mandatory in postgraduate programs	✓ Agree	Disagree	Neutral	I don't know
3. All investigators of human and animal studies should have training in research ethics	✓ Agree	Disagree	Neutral	I don't know
4. Not all participants comprehend research projects well. Accordingly, there is no need to provide them with details	Agree	✓ Disagree	Neutral	I don't know
5. There is no need to obtain informed consent to do research on blood samples already withdrawn for clinical tests	Agree	✓ Disagree	Neutral	I don't know
6. There should be a research ethics committee (REC) at each university	✓ Agree	Disagree	Neutral	I don't know
7. Only human subject research must be reviewed by an REC	Agree	✓ Disagree	Neutral	I don't know
8. Review by an REC would delay research projects and make it harder for the researcher to perform it	Agree	✓ Disagree	Neutral	I don't know
9. If there is a scientific committee for reviewing research, there is no need for an REC	Agree	✓ Disagree	Neutral	I don't know
10. Members of REC should be at least professors with high authority in the university	Agree	✓ Disagree	Neutral	I don't know
11. There is no need for child approval in research if they are less than 15 years as long as parents are consenting	Agree	✓ Disagree	Neutral	I don't know
12. Retrospective studies are exempt from informed consent	Agree	✓ Disagree	Neutral	I don't know
13. The researcher (by him/herself) can decide that no informed consent is needed if the research is a retrospective study (data already collected)	Agree	✓ Disagree	Neutral	I don't know
14. Informed consent should be always written	Agree	✓ Disagree	Neutral	I don't know

✓ Correct answers and/or what is considered good practices
